# Amino-Functionalized Polystyrene Nano-Plastics Induce Mitochondria Damage in Human Umbilical Vein Endothelial Cells

**DOI:** 10.3390/toxics10050215

**Published:** 2022-04-25

**Authors:** Yiqi Fu, Mengqi Fan, Liwang Xu, Hui Wang, Qinglian Hu, Yuanxiang Jin

**Affiliations:** College of Biotechnology and Bioengineering, Zhejiang University of Technology, Hangzhou 310032, China; 201906021901@zjut.edu.cn (Y.F.); 201706021824@zjut.edu.cn (M.F.); 2112005103@zjut.edu.cn (L.X.); 2112005028@zjut.edu.cn (H.W.)

**Keywords:** polystyrene nanoparticles, HUVEC, oxidative stress, mitochondria

## Abstract

As emerging contaminants, nano-plastics have become a major cause for concern for their adverse effects on the ecosystem and human health. The nano-sized properties of nano-plastics enable their exposure risks to humans through the food chain or other ways. However, the fate and adverse impact of nano-plastics on the human cardiovascular system are lacking. In this regard, the human umbilical vein endothelial cell line HUVEC was applied as a cell model to investigate the biological effects of noncharged polystyrene nano-plastics (PS NPs) and amino-functionalized nano-plastics (NH_2_-PS NPs). The positively charged PS NPs exhibited higher cytotoxicity to HUVEC, as evidenced by the decreased cell viability, enhanced ROS generation, and decreased mitochondria membrane potential triggered by NH_2_-PS NPs. Importantly, RT-PCR analysis revealed that NH_2_-PS NPs dysregulated the mitochondrial dynamics, replication, and function-related gene expression. This study demonstrated that NH_2_-PS NPs presented higher risks to endothelial cells than non-charged nano-plastics by interfering with mitochondria, which supported the direct evidence and expanded the potential risks of PS NPs.

## 1. Introduction

The increasing accumulation of plastic wastes in the environment has become a major cause for concern because of their adverse effects on the ecosystem and human health [1,2,3]. UV-exposure, and external and biological degradation were reported to be responsible for the breakdown of plastics into micro-particles <5000 nm in diameter, and further into nanoparticles (NPs) <100 nm in diameter [4,5]. As emerging contaminants, nano-plastics refer to plastic particles with sizes ranging between 1 nm and 1 μm [6]. Due to their nano-size fraction, they may be more extensively distributed and hazardous than larger-sized particles are [7,8,9]. Increasingly, nano-plastics have become the newest focus of the problematization of plastics in the environmental arena. Recently, new experimental regimes have specifically focused on plastic exposures to assess potential adverse effects to nano-plastics, including potentially negative impacts on low-trophic marine fauna, affecting liver functionality, neurotoxicity, and intestinal inflammation [10,11,12,13].

Particle size plays a crucial role in interactions with organisms and cells. In this perspective, nano-sized plastics can be easily taken up into cells and cross biological barriers, which lead to systemic uptake occurring and finally becoming enriched in higher organisms through the food chain [14,15,16]. Consequently, the risk assessing of nano-plastics has also extended into the area of human health in relation to their accumulation in the food chain. Due to their nano-dimensions, it is possible that nano-plastics could enter into the circulatory system and accumulate in major organs. To date, in vitro toxicity studies of nano-plastics have mainly focused on gastrointestinal cells, respiratory tract cells, liver cells, and immunity cells [17,18,19,20]. Endothelial cells line the lumen of all blood vessels, which play essential roles in nutrition, waste transportation, and cell movement [21]. The function of endothelial cells affects the blood vessel integrity and is associated with various diseases, such as cancer and inflammation [22]. Despite the concern about the toxicity of nano-plastics in humans, the information regarding their cytotoxic effects in human endothelial cells is lacking.

It has been recognized that the size and surface charge functionalization of nanoparticles influence their biological fate [23,24,25]. Due to the large surface area of nano-plastics, nano-plastics may adsorb and enrich many different materials, such as toxic pollutants, river natural organic matter, and positively charged minerals [26,27]. To date, polystyrene (PS) nanoparticles models with different sizes and controlled surface functionalization mimicking the nano-plastics formed during the natural degradation of plastic debris were used to evaluate their biological effects [28,29]. Although it has been suggested that positively charged nanoparticles are potentially more hazardous than negatively charged ones, little is known about the biological effects of nano-plastics with different charges to human vascular endothelial cells.

**Scheme 1:**
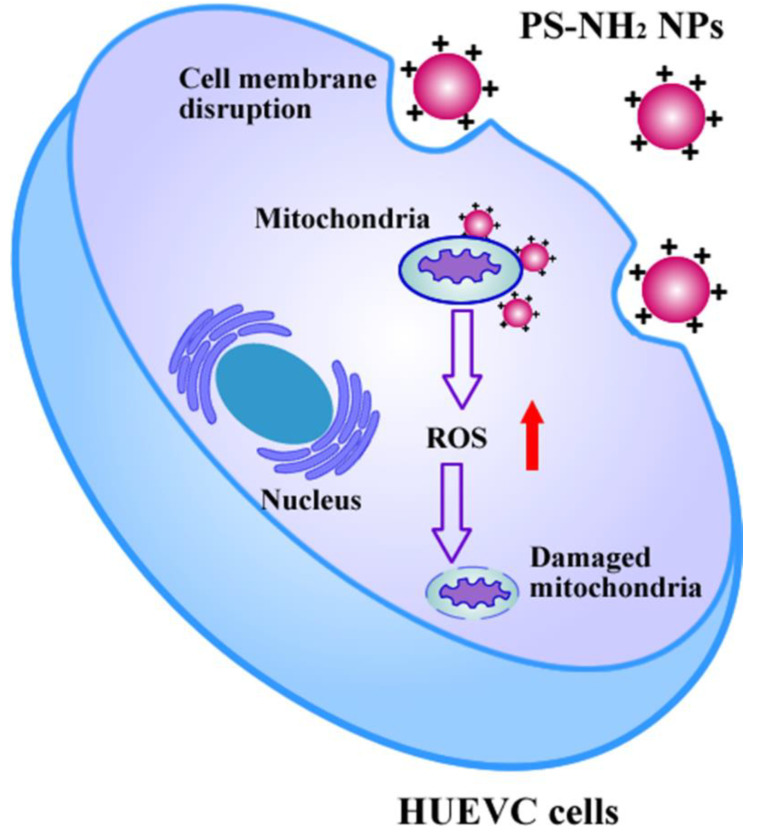
Schematic illustration of biological interaction of NH2-PS NPs with HUVEC.

In this contribution, the human umbilical vein endothelial cell line HUVEC was applied as a cell model to investigate the biological effects of noncharged polystyrene nano-plastics (PS NPs) and amino-functionalized nano-plastic (NH_2_-PS NPs). As indicated in Scheme 1, due to the negatively charged cell membrane, the positively charged PS-NH_2_ NPs could facilitate the cellular uptake and perturb cell membrane integrity. Subsequently, the internalized NH_2_-PS NPs could interact with the mitochondria of HUVEC, inducing ROS generation and mitochondria membrane potential decrease. Importantly, RT-PCR analysis revealed that NH_2_-PS NPs dysregulated the mitochondrial dynamics, replication, and function-related gene expression. This study aimed to understand the cellular fate and toxicity of PS NPs and NH_2_-PS NPs to HUVEC, which may help replenish the potential risks of nano-plastics to mammals.

## 2. Methods and Materials

### 2.1. Materials

PS NPs and NH_2_-PS NPs were acquired from Sigma–Aldrich (Sigma-Aldrich, St. Louis, MI, USA). 3-(4,5-dimethylthiazol-2-yl)-2,5-diphenyl tetrazolium bromide (MTT), 2′,7′-dichlorodihydrofluorescein di-acetate (DCF), JC1, and LDH Detection Kit were purchased from Beyotime Institute of Biotechnology (Jiangsu, China). TRIzol reagent was acquired from Takara (Takara Biochemicals, Dalian, China). The ReverTra Ace^®^ qPCR RT Kit and SYBR^®^ Green Realtime PCR Master Mix were from Toyobo (Tokyo, Japan). All oligonucleotide primers were synthesized in Sangon Biotech (Sangon, Shanghai, China).

### 2.2. Nano-Plastics Characterization

The particle size and zeta potential of PS NPs and NH2-PS NPs were determined at 25 °C by dynamic light scattering (DLS) on the Zeta-sizer 3000 (Malvern Instruments, Worcestershire, UK). The average of 3 measurements of 50 cycles was used as a numerical value of zeta potential. The morphology of PS NPs and NH2-PS NPs was evaluated by scanning electron microscopy (SEM).

### 2.3. Cell Culture

The HUVEC cell line was obtained from American Type Culture Collection (ATCC) and cultured under standard cell culture conditions in RPMI 1640 (Invitrogen, Carlsbad, CA, USA), supplemented with 10% heat-inactivated FBS (Gibco, St. Louis, MI, USA) and 1% penicillin-streptomycin (Thermo Scientific, Waltham, MA, USA). Before experiments, the cells were pre-cultured until confluence was reached.

### 2.4. Cell Viability Measurement

An MTT assay was applied to determine the cytotoxicity of nano-plastics. HUVEC cells were seeded in 96-well plates at a density of 10,000 cells per well. After 24 h of incubation, the medium was replaced with 5, 10, 15, 20, and 25 μg/mL of PS NPs and NH_2_-PS NPs and further incubated at 37 °C for 12 or 24 h. Then, the cells were washed with PBS and 0.5 mg/mL of MTT working solution was added and maintained at 37 °C for 4 h. Finally, each well was replaced with 100 μL of DMSO to dissolve the formazan crystals, and the absorbance was measured at 490 nm using a microplate reader (Themo Multiscan MK3, Waltham, MA, USA). The untreated cells served as the control and their viability was set as 100%.

Lactate dehydrogenase (LDH) release was evaluated to acquire additional information about the cytotoxicity of the nanoparticle. Briefly, after exposure to 10 and 20 μg/mL of PS NPs and NH_2_-PS NPs for 24 h, LDH analysis was carried out according to the manufacturer’s instructions of the LDH Cytotoxicity Assay Kit (Beyotime, Shanghai, China). The absorbance values were read at 490 nm in a microplate reader (Themo Multiscan MK3, Waltham, MA, USA).

### 2.5. Living and Dead Cell Staining

HUEVC cells (50,000 cells per well) were seeded in 24-well plates and cultured at 37 °C in a 5% CO_2_ atmosphere for 24 h. After the cells were incubated with 10 and 20 μg/mL of PS NPs and NH2-PS NPs for 24 h, the cells were washed with PBS buffer. Afterward, the cells were co-stained with Calcein-AM (green color for live cells) and propidium iodide (PI) (red color for dead cells) according to the manusfacture’s instruction. We observed the results through a microscope (Oplenic Digital Camera, Nikon, Japan) after being washed three times with PBS.

### 2.6. Intracellular ROS Level Determination

The intracellular ROS generation was detected by 2′,7′-dichlorofluoresce in diacetate (DCFH-DA). DCFH-DA is nonfluorescent but switches to the highly fluorescent DCF when oxidized by intracellular ROS. For fluorescent imaging, HUVEC cells were seeded in 48-well plates at a density of 5 × 10^4^ cells per well and cultured for 18 h. Then, different concentrations of PS NPs and NH_2_-PS NPs solutions were added and incubated with cells for a further 24 h. After exposure, cells were washed thrice with PBS and were stained with DCFH-DA (10 μM) for 30 min at 37 °C. Afterward, the cells were imaged by a fluorescence microscope (Nikon PCS603-07) with an OPLENIC industrial digital camera.

For quantitative analysis of ROS, HUVEC cells were seeded in 12-well plates at a density of 10 × 10^4^ cells per well. Cells were treated with the different concentrations of PS NPs and NH_2_-PS NPs for 48 h and were stained with DCFH-DA (10 μM) for 30 min at 37 °C. Then, the cells were washed, trypsinized, and re-suspended in PBS. Flow cytometry measurements were conducted using Cyan-LX (Dako Cytomation, California, USA)). The mean fluorescence was determined by counting 10,000 events.

### 2.7. Mitochondria Membrane Potential Detection

The mitochondrial membrane potential (MMP) was measured by JC-1 staining, which can accumulate in active mitochondria. Briefly, HUVEC cells were seeded in 12-well plates and grown for 24 h. After the cells were treated with PS NPs and NH_2_-PS NPs for 24 h at 37 °C, the cells were washed and stained with a 10 μg/mL JC-1 solution for 30 min at 37 °C. Then, the cells were imaged by the fluorescence microscope (Nikon PCS603-07) with the OPLENIC industrial digital camera.

### 2.8. Quantitative Real-Time PCR Analysis

The mRNA expression level of mitochondria function-related genes were measured by semi-quantitative real-time PCR analysis (RT-qPCR). HUVEC cells were seeded at an initial density of 2.5 × 10^5^ cells/well in 6-well plates and after treatment with different concentrations of PS NPs and NH_2_-PS NPs for 24 h. The cells were collected to isolate RNA with TRIzol reagent (Takara Biochemicals, Dalian, China) according to the manufacturer’s protocol. Subsequently, first-strand cDNA was synthesized using the ReverTra Ace qPCR RT Kit (Toyobo, Tokyo, Japan). The quantification of cytokines expression was employed by using the SYBR green system (Toyobo, Tokyo, Japan). The sequences of primers for qPCR are listed in Table 1. The PCR parameters were as follows: 95 °C for 1 min, 40 cycles of 95 °C for 15 s, and 60 °C for 1 min. The gene expression was analyzed with the 2^−^^△△Ct^ method and normalized to the housekeeping gene Gapdh as the endogenous reference.

### 2.9. ATP Activity Assay

The relative cellular ATP activity was measured by using a firefly luciferase-based ATP assay kit (promega). Briefly, HUEVC cells were seeded in 96-well plates for 24 h to achieve 80% confluence. After the cells were incubated with 10 and 20 μg/mL of PS NPs and NH2-PS NPs for 24 h, the cells were washed with PBS buffer and 100 μL of PBS was further added to the well. Another 100 μL of the diluted kit reagent was mixed with the cells for 2 min. The luminance (relative luminescence units, RLU) was measured by a micro-plate reader (Themo Multiscan MK3, Waltham, MA, USA).

### 2.10. Statistics

All data were expressed as means ± standard error of the mean (SEM). Statistical analysis was performed using GraphPad Prism 5 (GraphPad Software, La Jolla, CA, USA). Statistical differences between replicates were tested using one-way analysis of variance (ANOVA) by StatView 5.0.1 (Cary, NC, USA).

## 3. Results and Discussion

**Figure 1:**
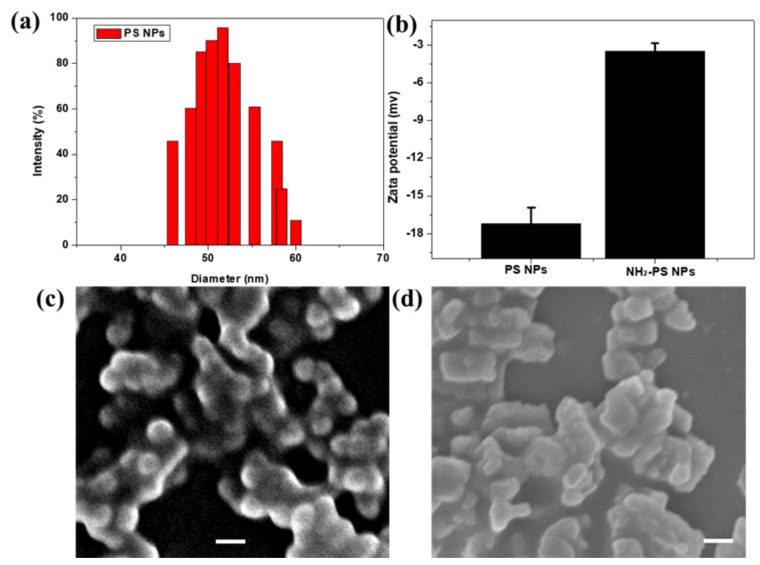
Hydrodynamic diameter of PS NPs (**a**) and zeta potential of PS NPs and NH_2_-PS NPs determined by dynamic light scattering (DLS) (**b**); scanning electron microscopy (SEM) images of PS NPs (**c**) and NH_2_-PS NPs (**d**). The scale bars represent 100 nm.

The nanoparticle–cell interaction and fate of nanoparticles are mediated by different factors, among which size, surface charge, composition, and cell type play an important role. During the degradation process, plastics can undergo a considerable structural transformation such as surface functionalization [30]. In our study, PS NPs and NH_2_-PS NPs were chosen to model NPs to investigate their biological toxicity to HUEVC. Considering that nanoparticle characterization is the primary part for nanoparticle toxicity screening, the particle size and zeta potential of PS and NH_2_-PS NPs were first examined by dynamic light scattering (DLS). As shown in Figure 1a,b, the hydrodynamic size of PS NPs was 50 nm and the zeta potentials were –17.6 and –5.6 mv for PS and NH_2_-PS NPs, respectively, according to DLS measurements. As shown in Figure 1c,d, PS and NH_2_-PS NPs exhibited homogeneous size distributions and the average primary particle sizes were 50 nm.

**Figure 2:**
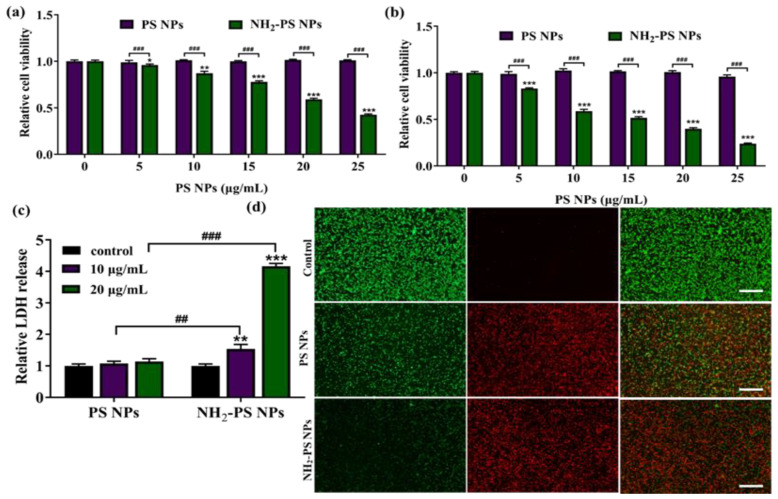
The effects of PS NPs and NH_2_-PS NPs on the viability of HUVEC after exposure for 12 h (**a**) and 24 h (**b**); the effects of PS NPs and NH_2_-PS NPs on the LDH release of HUVEC after 24 h (**c**); and the live/dead staining of HUVEC cells after incubation with 20 μg/mL of PS NPs and NH_2_-PS NPs for 24 h (**d**). Error bars correspond to standard deviations. *n* = 5. * *p* < 0.05, ** *p* < 0.01, *** *p* < 0.001 compared with control group, ## *p* < 0.01, ### *p* < 0.001 compared between PS NPs and NH_2_-PS NP. The scale bars represent 100 μm.

Previous studies have revealed that amine-functionalized PS NPs with a size of 50 nm exhibited a much obvious increased cellular uptake in HepG2 cells [17], alveolar cells [19], and SNU-1 [31] than PS NPs did, indicating that surface functionalization facilitated the internalization process of PS NPs. The internalization of PS NPs is strongly related to their potential risks. The cytotoxicity is an important indicator to evaluate the risk of the nano-plastics. Thus, we initially examined the extent of cytotoxicity caused by the PS NPs and PS-NH_2_ NPs by MTT assay. As shown in Figure 2a,b, after 12 h and 24 h of treatment, negligible cytotoxicity was observed in PS NPs-treated groups within the tested concentrations, while PS-NH_2_ NPs exhibited concentration- and time-dependent cytotoxicity. At the concentration of 20 μg/mL, the cell survival rate was decreased to less than 30%. Various studies have reported that the cytotoxicity of plastics might vary in different cell lines and with different surface functionalization. For example, 100 μg/mL of PS-NH_2_ with a size of 60 nm was reported to cause 75% inhibition of cell viability in human BEAS-2B cells in 16 h [32]. In addition, PS-NH_2_ at a concentration of 50 μg/mL was reported to exhibit a higher inhibition of cell viability in HepG2 cells than PS-COOH [17]. A recent study revealed that 50 nm aminated particles showed the greatest toxicity within 1 h of treatment in SUN-1 cells (≥7.5 μg/mL) [31]. We and others’ work emphasized that 50 nm amine-functionalized PS NPs were more toxic than PS NPs were, perhaps because of their positive charge more easily passing through the phospholipid bilayer of the cell membrane and causing membrane damage to a greater extent than noncharged counterparts.

LDH release is regarded as an important indicator of cell membrane integrity. Cationic particles are known to cause great extents of lipid bilayer disruption [33]. LDH analysis was carried out according to the manufacturer’s instructions of the LDH Cytotoxicity Assay Kit and the absorbance values were read at 490 nm in a microplate reader. As expected, upon treatment of 20 μg/mL of PS-NH_2_ NPs, the LDH release was significantly increased, as compared to control or PS NPs, indicating that the positively charged PS-NH_2_ NPs could damage the cell membrane integrity of HUVEC (Figure 2c). The live/dead staining was applied to visualize the effects of PS NPs and NH_2_-PS NPs on HUVEC cells viability. As shown in Figure 2d, a strong green fluorescence signal was observed in the control group. As compared to 20 μg/mL of PS NPs, the significantly decreased green fluorescence and bright red fluorescence reflect that many more HUVEC cells were dead in the PS-NH_2_ NPs-treated groups. This result was consistent with the results of MTT.

**Figure 3:**
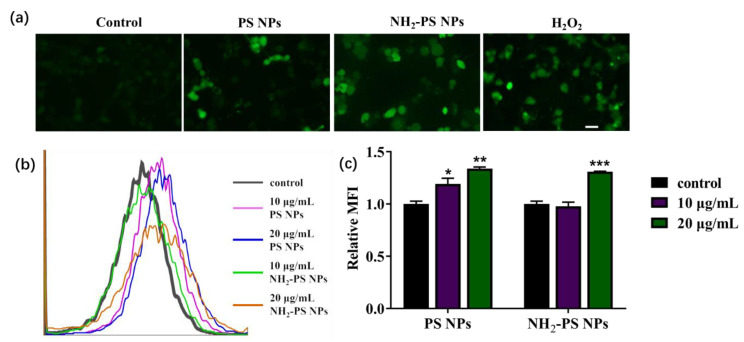
The effects of PS NPs and NH2-PS NPs on ROS production in HUVEC. The fluorescent images (**a**) and flow cytometry analysis (**b**) of HUVEC cells upon treatment with 20 μg/mL of PS NPs and NH2-PS NPs, co-stained with DCFH-DA. The mean fluorescence intensity (MFI) was analyzed by FlowJo and made into the graph (**c**). The scale bar represents 20 μm. N = 4, * *p* < 0.05, ** *p* < 0.01, *** *p* < 0.001 compared with control group.

To understand the cell oxidative stress caused by PS NPs and PS-NH2 NPs, we monitored the intracellular reactive oxygen species (ROS) level in HUVEC cells by 2′,7′-dichlorofluorescein diacetate (DCFH-DA) staining. DCFH-DA is nonfluorescent but switches to the highly fluorescent DCF when oxidized by intracellular ROS. As shown in Figure 3a, as compared to the control, the brighter green fluorescence was detected in the 20 μg/mL PS NPs- and NH_2_-PS NPs-treated HUVEC. Quantitative measurement of ROS generation was conducted by flow cytometry. As shown in Figure 3b, the peak of the fluorescence was increased when treated with 10 and 20 μg/mL of PS NPs and NH_2_-PS NPs. From the mean fluorescence intensity of flow cytometry, it was found that there was no difference between PS NPs and NH_2_-PS NPs (Figure 3c). ROS are recognized as important initiators and mediators of cell death. It is well known that nanoparticles are enabled to induce cellular toxicity via elevated ROS [34]. However, a low-to-moderate increase in intracellular ROS serves as a secondary messenger, which have proliferation properties [35]. Our study revealed that, as compared to NH_2_-PS NPs, 10 μg/mL of PS NPs exhibited a lower cytotoxicity but higher ROS level, suggesting that PS NPs with different changes may induce cytotoxicity via a different mechanism. The higher cytotoxicity of NH_2_-PS NPs may be ascribed to their direct damage to the cell membrane, which is evidenced by the LDH release results.

**Figure 4:**
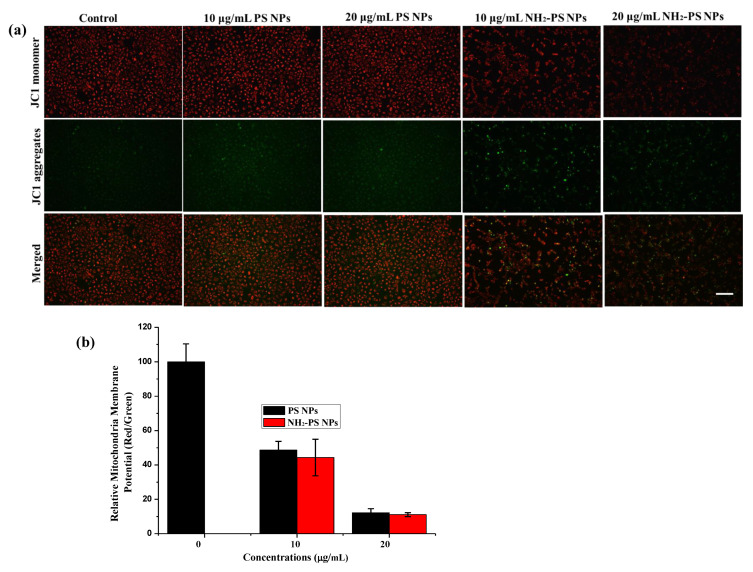
The fluorescent images (**a**) of HUVEC with treatment of 10 and 20 μg/mL of PS NPs and NH2-PS NPs on mitochondrial membrane potential in HUVEC. The effects of PS NPs and NH_2_-PS NPs on mitochondrial replication in HUVEC. The mean fluorescence intensity was quantified by Image J software (**b**). The images share the same scale bar of 100 μm.

Considering that increased intracellular ROS may attack mitochondria and lead to mitochondrial dysfunction, the mitochondrial membrane potential in HUVEC treated by PS NPs and NH_2_-PS NPs was examined by JC-1 staining and the fluorescence was monitored by fluorescent microscopy. The red fluorescence represents JC-1 aggregates, indicating high mitochondrial membrane potential, while the green fluorescence represents the JC-1 monomer, indicating low mitochondrial membrane potential. From Figure 4a, the bright red fluorescence in the control suggested the healthy state of control cells. However, NH_2_-PS NPs exposure significantly downregulated the mitochondrial membrane potential in HUVEC cells, which was manifested as a decrease in red fluorescence and an increase in green fluorescence. The quantification results also confirmed the decreased mitochondria membrane potential upon treatment of PS NPs and NH_2_-PS NPs. In the present study, we demonstrated that NH_2_-PS NPs could cause a greater change in mitochondria membrane potential than PS NPs. The positive changes of NH_2_-PS NPs facilitated their absorption to the mitochondria membrane and direct attacking of mitochondria, leading to a depolarized mitochondria membrane potential. As indicated in Figure 4b, the relative ATP activity in the 10 and 20 μg/mL NH_2_-PS NPs-treated HUEVC cells decreased to 82% and 53% of the control, respectively.

**Figure 5:**
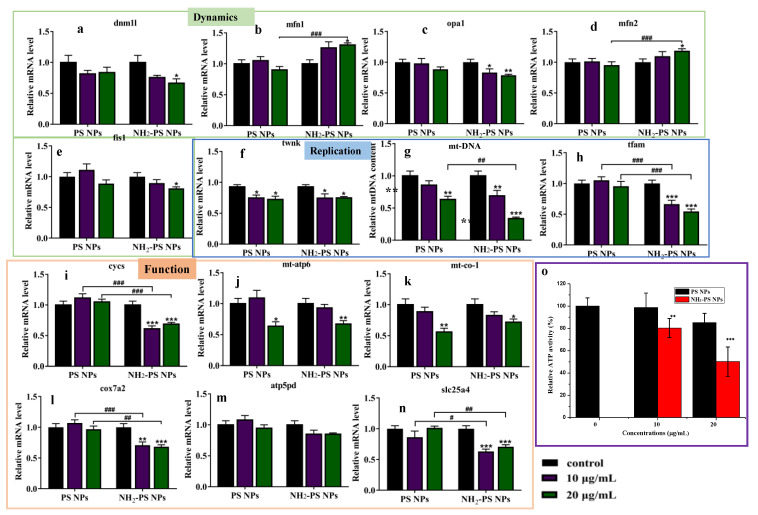
The RT-qPCR analysis of PS NPs and NH_2_-PS NPs on the relative expression of mitochondrial dynamics-related genes *dnm1l* (**a**), *mfn1* (**b**), *opa1* (**c**), *mfn2* (**d**), and *fis1* (**e**); on the relative expression of mitochondria replication-related genes *twnk* (**f**), *mt-DNA* (**g**), and *tfam* (**h**); on the relative expression of mitochondria function-related genes *cycs* (**i**), *mt-atp6* (**j**), *mt-co-1* (**k**), *cox7a2* (**l**), *atp5pd* (**m**), and *slc25a4* (**n**). Error bars correspond to standard deviations. *n* = 4. * *p* < 0.05, ** *p* < 0.01, *** *p* < 0.001 compared with control group, # *p* < 0.05, ## *p* < 0.01, ### *p* < 0.001 compared between PS NPs and NH_2_-PS NP. Effect of PS NPs and NH_2_-PS NP on the relative activity of HUEVC cells (**o**), *n* = 4. ** *p* < 0.01, *** *p* < 0.001 compared with control group.

The mitochondria membrane potential plays an essential role in maintaining mitochondria function; thus, we first investigated the mitochondrial dynamics-related gene expression by semi-quantitative real-time PCR analysis (RT-qPCR) after exposure of PS NPs and NH_2_-PS NPs. The morphology of mitochondria is determined by the balance between mitochondrial fusion and division [36]. Thus, the expression levels of genes related to mitochondrial fusion and fission were first detected. As indicated in Figure 5, PS NPs treatment had limited effect on the expression of mitochondrial fusion and fission-related genes in HUVEC cells. However, upon treatment of NH_2_-PS NPs, the mRNA expression level of *mfn2* was significantly increased as compared to the control, which suggested that NH_2_-PS NPs exhibited the potential to promote the outer membrane fusion of mitochondria. In addition, mRNA expression levels of *fis1*, *dnm11*, and *opa1* in NH_2_-PS NPs-treated groups were significantly decreased, which are associated with mitochondria division and fusion of the inner membrane. These results suggested that NH_2_-PS NPs could disrupt the balance between mitochondrial fusion and division.

In addition, the mitochondria replication-related genes expression was quantified by RT-qPCR. After exposure to NH_2_-PS NPs, the mitochondrial DNA content decreased significantly at 10 and 20 μg/mL, and the mitochondrial dynamics-related gene expression was decreased significantly, especially *tfam* and *twnk* (Figure 5f,h). Finally, we examined the effects of PS NPs and NH_2_-PS NPs on mitochondrial function from the expression levels of ATP synthesis-related genes (*atp6h*, *slc25a4*, *atp5h*) and respiratory chain-related genes (*cycs*, *co-1*, *cox7a2*). Obviously, ATP synthesis-related atp6h and mitochondrial respiratory chain-related gene co-1 exhibited a decreased expression upon exposure to 20 μg/mL of PS NPs. However, upon treatment of 10 μg/mL of NH_2_-PS NPs, the behavior of mitochondria function-related genes, especially *slc25a4*, *cycs,* and *cox7a2*, significantly decreased. In the 20 μg/mL NH_2_-PS NPs-treated groups, the examined genes including *cycs*, *mt-co-1*, *cox7a2,* and *slc25a4* showed a significant downward tendency (Figure 5i,k,l,n), indicating the potential risks of NH_2_-PS NPs to mitochondria function of HUVEC. Mitochondria are vital subcellular organelles to eukaryotic cells, which play valuable roles in energy production, ROS generation, and cellular signaling [37]. Therefore, we assessed the effects of PS NPs on the ATP production capacity. As indicated in Figure 5o, the relative ATP activity in the 10 and 20 μg/mL NH_2_-PS NPs-treated HUEVC cells decreased to 80% and 50% of the control, respectively. Our results revealed that NH_2_-PS NPs could dysregulate the mitochondrial dynamics, mitochondrial replication, and mitochondrial function-related genes mRNA expression, and downregulate ATP production capacity, indicating the impairment of mitochondria function.

In summary, our study demonstrated that positively charged PS NPs behave more toxic to HUVEC cells, as evidenced by the dosage-related cell cytotoxicity. In addition, NH_2_-PS NPs result in oxidative stress and induce damage to the mitochondria membrane potential, which are essential factors to maintain the mitochondria function. Moreover, NH_2_-PS NPs exhibited a stronger ability to dysregulate the mitochondrial dynamics, replication, and function-related genes expression. Our results highlighted the importance of surface charge on the biological interaction of PS NPs.

## Figures and Tables

**Table 1:** Sequences of primer pairs used in the real-time quantitative PCR.

Gene	Sequence
gapdh	Forward 5′-GCACCGTCAAGGCTGAGAAC-3′
	Reverse 5′-TGGTGAAGACGCCAGTGGA-3′
12S rRNA (mtDNA)	Forward 5′-TAACCCAAGTCAATAGAAGCC-3′
	Reverse 5′-CTAGAGGGATATGAAGCACC-3′
tfam	Forward 5′-ATGGCGTTTCTCCGAAGCAT-3′
	Reverse 5′-TCCGCCCTATAAGCATCTTGA-3′
twnk	Forward 5′-GGATCGCAGCTCAAGACTACA-3′
	Reverse 5′-GGTGAATGACCAGTGTCACAT-3′
dnm1l	Forward 5′-CGACTCATTAAATCATATTTTCTCATTGTCAG-3′
	Reverse 5-TGCATTACTGCCTTTGGCACACT-3′
mfn1	Forward 5′-CTGAGGATGATTGTTAGCTCCA-3′
	Reverse 5′-CAGGCGAGCAAAAGTGGTAGC-3′
mfn2	Forward 5′-TGGACCACCAAGGCCAAGGA-3′
	Reverse 5′-TCTCGCTGGCATGCTCCAC-3′
opa1	Forward 5′-TGGTGCTGTTTCAGTCCAAG-3′
	Reverse 5′-AGCCTCACTGTCGTTTTTGC-3′
fis1	Forward 5′-CCTGGTGCGGAGCAAGTACAA-3′
	Reverse 5′-TCCTTGCTCCCTTTGGGCAG-3′
atp6	Forward 5′-CTGTTCGCTTCATTCATTGC-3′
	Reverse 5-AGTCATTGTTGGGTGGTGATT-3′
atp5h	Forward 5′-GCTGGGCGAAAACTTGCTCTA-3′
	Reverse 5′-CCAGTCGATAGCTGGTGGATT-3′
slc25a4	Forward 5′-ATCACGCTTGGAGCTTCCTAA-3′
	Reverse 5′-TGCTTCTCAGCACTGATCTGT-3′
co-1	Forward 5′-CGTTGTAGCCCACTTCCACT-3′
	Reverse 5′-TGGCGTAGGTTTGGTCTAGG-3′
cox7a	Forward 5′-CTCGGAGGTAGTTCCGGTTC-3′
	Reverse 5′-TCTGCCCAATCTGACGAAGAG-3′
cycs	Forward 5′-CTTTGGGCGGAAGACAGGTC-3′
	Reverse 5′-TTATTGGCGGCTGTGTAAGAG-3′

## Data Availability

The data presented in this study are available on request from the corresponding author. The data are not publicly available due to privacy.
